# Plasma Proteomic Profiling of Neurodegenerative Proteins in Autosomal Dominant Alzheimer's Disease with *PSEN1* Mutations Relative to Sporadic Alzheimer's Disease and Cognitively Unimpaired Controls

**DOI:** 10.1002/alz70856_106861

**Published:** 2026-01-08

**Authors:** Alexandra M Izydorczak, Marissa F Farinas, Xuemei Zeng, Anuradha Sehrawat, William E Klunk, Tharick A Pascoal, Ann D Cohen, Victor L. Villemagne, Dana L Tudorascu, C. Elizabeth Shaaban, Jennifer H Lingler, Beth E. Snitz, Eric E Abrahamson, Sarah B Berman, Julia K. Kofler, Milos D. Ikonomovic, M. Ilyas Kamboh, Oscar L Lopez, Thomas K Karikari

**Affiliations:** ^1^ University of Pittsburgh, Pittsburgh, PA, USA; ^2^ University of Pittsburgh School of Medicine, Pittsburgh, PA, USA; ^3^ University of Pittsburgh Alzheimer's Disease Research Center (ADRC), Pittsburgh, PA, USA; ^4^ UPMC, Pittsburgh, PA, USA

## Abstract

**Background:**

A major pathological hallmark of Alzheimer's disease (AD) is plaque deposition of amyloid‐β (Aβ) peptide which is cleaved from a larger Aβ precursor protein (APP). One of the most common causes of autosomal dominant AD (ADAD) are mutations in the *PSEN1* gene, encoding a component of γ‐secretase, which alter APP processing and increase the production and aggregation potential of Aβ. However, the impact of *PSEN1* mutations on blood biomarker levels of neurodegeneration‐related proteins is largely unexplored.

**Method:**

We applied a novel Nucleic Acid Linked Immuno‐Sandwich Assay (NULISA), the NULISAseq CNS Disease Panel, to quantify 127 key neurodegenerative proteins in plasma samples from a cohort consisting of ADAD cases with *PSEN1* mutations (*n* = 6), neuropathologically diagnosed sporadic AD (sAD; *n* = 8), and cognitively unimpaired controls (*n* = 7). Normalized protein quantification (NPQ) was used as a proxy for protein levels. Statistical comparisons were performed using the Kriskal‐Wallis and Wilcoxon rank‐sum tests. Spearman correlation was employed to evaluate the association between NULISA and Single Molecule Array (SIMOA)‐based measurements.

**Result:**

The NULISA had high detectability (95.5% ± 13.6%) and reproducibility (median intra‐plate coefficient of variation [CV]=6.3%). NULISA and SIMOA correlated robustly for *p*‐tau181, *p*‐tau217, GFAP, NfL, and Aβ42 (Spearman coefficients ρ range 0.721 to 0.947), but less so for Aβ40 (ρ=0.302). Total‐tau (MAPT), *p*‐tau181, *p*‐tau217, and *p*‐tau231 were among the proteins most significantly different across the sample groups (*p* values range 0.001 to 0.003), followed by NfL, NfH, CCL2, GFAP, and PDGFRB (*p* values range 0.005 to 0.046). Several targets, including NfH (*p* = 0.001), CCL11 (*p* = 0.013), CHIT1 (*p* = 0.021), PDGFRB (*p* = 0.029), PTN (*p* = 0.029), and CNTN2 (*p* = 0.043), showed statistically significant differences between ADAD and sAD.

**Conclusion:**

Proteomic profiling of key neurodegenerative proteins in plasma revealed significant differences among the sample groups and include identified targets that can distinguish ADAD from sAD. Further validation with larger cohorts is needed to confirm these findings.

